# Anatomy

**Published:** 1877-01

**Authors:** 


### I. Anatomy.

1. *A Living Specimen of Human Pygomelia—or a Woman with Three Legs.* [Di un caso vivente di Pygomelia umana osservato in Roma.] Angelo Incoronato. (*Extract from the proceedings of the Royal Medico-Chirurgical Academy of Naples*, 1876.)

Mad. Blanche, when examined by the author, was twenty-six years old. She stated that her mother had had children both older and younger than herself, all of whom were normal in organization, and enjoyed perfect health. As in most of such cases, the pregnancy preceding the birth of Blanche had presented no anomaly, and the labor was not unusually painful or tedious.

In person, the subject of this sketch was found to be slightly below the average stature of females; her countenance had a happy and agreeable expression, which did not denote physical suffering; her head was rather large, the face presenting a slight reentrant angle between the forehead and the chin. The tint of the skin was that of the delicate "pink and white" complexion; the hair abundant; the lips full, red and expressive; the neck well turned and white; the breasts of moderate size and regular conformation, provided also with nipples and areolæ which resembled those of a girl fifteen years old. But the pelvis was disproportionately broad, the distance between the two anterior superior iliac spinous processes being increased—the right on a lower level than that on the left, due to pelvic obliquity. In consequence, the abdominal integument was stretched like a tense drum-head, and the abdomen, instead of projecting, remained flattened and depressed. A single umbilical cicatrix appeared in the median line, the pubis being also single, though broad and of unusual extent. The genital organs were double—each that of the female—disposed, one set on each side of the axis of the body. The left vulvar aperture was the larger, and permitted the introduction of the index finger; that on the right would scarcely admit the little finger; both were surmounted by a growth of hair, more abundant on the left side. In each vulva appeared a clitoris and corresponding meatus urinarius with a circular hymen—the latter forbidding further exploration with the finger; and the author was not permitted to use the sound or any other instrument in his examination. The urine was simultaneously voided from the two urinary apertures; and it was further stated that the menstrual blood was discharged from the two vulvæ simultaneously, and from each in about the same quantity.

Behind the left genital fissure, and at a normal distance, was found the sole anal aperture. Behind the right vulva there was no opening of any sort; but at a distance of about five centimeters, in a line passing upward to the dorsum of the ilium, was a cylindroid cutaneous appendix, about two centimeters long, insensible to the touch, impervious, always flaccid, and never rigid or turgid. In a careful palpation of this appendix, the fingers detected the origin of two filaments or small cords, having the feel of spermatic cords enlarged by circocelé, which, even upon rude handling, did not give rise to a painful or disagreeable sensation. On the promontory of the pubis, but much removed to the right side, was a third breast — abdominal or better inguinal in site — provided with nipple and areola quite insensible to the touch, becoming turgid on gentle friction, but without producing such reflex sensations as those effected by irritation of the thoracic mammary glands. To complete the history of the examination of the trunk, it is only necessary to add that the spine was normal from the cervical to the lumbar vertebræ, but the sacro-coccygeal region was much larger than usual, and terminated below in two diverging and completely ossified points. At the prolongation of the right coccygeal apex, a large pyriform tumor was situated, its greater diameter in continuity with the vertebral axis. This tumor was insensible to any external impression, and possessed the elasticity of a lipoma.

Two limbs supported the trunk, while a third, which was situated between the former, did not aid either in the retention of position or progression; it was affected with partial anchylosis of the knee-joint, and could not be placed upon the ground. This angular semi-anchylosis only permitted a *diminution* of the angle of 90° at the knee, without allowing its increase — hence the heel could touch the nates but not the floor. It did not appear to have a true articulation with the trunk, but rather to be implanted upon it like a tumor; nor could it be extended or moved volun tarily, as it was necessary to use the hand in bringing it either backward or forward. In its superior (or thigh) segment, it was quite large, and through its soft parts the fingers could detect a well developed bone, corresponding neither to that of an athletic man nor of a young girl. The integument covering the condyles of the knee was not provided with sufficient fat to give it a rounded form; it was therefore nodose in contour, and the leg also was thin and lean, as if deprived of its soft parts. The skin scarcely sufficed to cover the bone, which was quite large — not double — but the representative of an enormous misshapen tibia. The attached foot, which was affected with varus, was flexed upon the leg, and held in position by a strong anchylosis; it was emaciated, and terminated in five clubbed and twisted toes, the great toe pointing to the left heel; therefore the foot was regarded as belonging to a right leg. Its temperature was decidedly lower than that of the other limbs, and even lower than that of the tumor and cutaneous appendix described above.

The other two limbs which sustained the body were somewhat irregular and differed, each from the other. The right was much the more shapely; its muscular masses were clothed with adipose tissue and normal integument, and its rounded form differed from that of the rest of the body, especially in its upper portion. The foot terminated in five toes, the great toe pointing inward, and exhibiting no deformity. Yet the great trochanter of the femur was excessively developed, and the articular head of the bone, instead of being enclosed in an acetabulum, seemed to rest in a pseudo-articular cavity, constituted by the external iliac fossa. The left limb was much smaller and shorter than that just described; and this fact, in connection with the abnormal right coxo-femoral articulation, seemed to the author to explain the obliquity of the pelvis, which brought the anterior superior spines of the ilium into different planes. The leg was excessively small, disproportioned to the size of the thigh, which had a more regular conformation. The foot of this limb also was varus, as that of the median extremity, and turned inward to such an extent that the point of the foot was directed posteriorly. When touching the ground it rested exactly upon its internal border, this position having produced a dense callosity over the cuboid bone by the consequent pressure in that region. The foot terminated in five distorted and anchylosed toes, the great toe pointing inward. This limb differed also from the others in the extent to which it was capable of being removed from and approximated to the others — a circumstance readily seen when the subject walked with feet exposed.

The author critically analyzes the phenomena described above, and finds that Mad. Blanche belongs to the family of monsters called *polimelici*, of the genus *pygomeles* or *epipygus;* that is, there existed two primitive beings, which became united by the sacrum and coccyx — one attaining its development through the phases of evolution, the other arrested in development, but still exhibiting duplicity of the sexual organs, a supernumerary limb and other anomalies of the body — a species of monstrosity rare in mammals, especially in man, though frequently encountered in birds.

After sketching the probable course of development from the germinative areæ, the author proceeds to show that there was a union of two pelves; four ilia and two pubic and sacral bones; complete or partial rescrption of the two internal ilia; solidification of the two sacral and coccygeal parts; and hence no articulating surface for the third limb, whose muscles became consequently the subject of fatty degeneration and inertia. The median and left limbs thus corresponded to an individual formed on the left of the axis of fusion; the right to an imperfectly developed individual on that side of the axis. The feet of the two first named were both affected with varus; the last was the most normal in size and shape. On the left also was the sole anal aperture and the best formed genitalia. On the right, it is true, the genitalia were not wanting, but the third breast, the cutaneous appendix and the pyriform tumor, all showed the irregular distribution and development of a primitive plastic material.

In all probability the pyriform tumor was the nucleus or rudiment of a left leg of the imperfectly developed individual on the right side. The cutaneous appendix could only be explained as an instance of exuberant plastic material, viciously disposed and directed. As to the internal genitals, the simultaneity of the emission of urine and menstrual blood might be explained by supposing single, double or bicorned uterus, as well as single or double bladder, for the unicity of the spinal cord and nervous center presiding over these functions, would, in any event, supply the explanation of this peculiarity.

